# Brain–Computer Interfaces for Human Augmentation

**DOI:** 10.3390/brainsci9020022

**Published:** 2019-01-24

**Authors:** Davide Valeriani, Caterina Cinel, Riccardo Poli

**Affiliations:** 1 Department of Otolaryngology, Massachusetts Eye and Ear, Harvard Medical School, Boston, MA 02114, USA; davide_valeriani@meei.harvard.edu; 2 Brain-Computer Interfaces and Neural Engineering Laboratory, School of Computer Science and Electronic Engineering, University of Essex, Wivenhoe Park, Colchester CO4 3SQ, UK; ccinel@essex.ac.uk

The field of brain–computer interfaces (BCIs) has grown rapidly in the last few decades, allowing the development of ever faster and more reliable assistive technologies for converting brain activity into control signals for external devices for people with severe disabilities. In recent years, however, the scope of BCIs has been extended from assistive technologies to neuro-tools for human cognitive augmentation for everyone. For instance, novel applications of BCIs have been proposed, enabling people to go beyond human limitations in sensory, cognitive, and motor tasks [1,2,3,4]. These include new and exciting paradigms, such as BCIs based on the brain activity of multiple people [5]. 

The aim of this special issue was to gather high-quality papers—including both reviews and reports on novel research—representative of the ongoing research in the area of BCIs for human cognitive augmentation. Twelve manuscripts were received through the open submission window, which went through a rigorous selection, peer review, and revision process, resulting in five papers being accepted for publication within the special issue. These papers are briefly described below.

One of the earliest BCI applications, the famous P300 matrix speller developed by Farwell and Donchin over three decades ago [6], provided a simple and practical way for restoring communication capabilities to the paralyzed. Since then, a large variety of spellers have been developed, which explore different paradigms, graphical user interfaces, neuroimaging techniques, and signals from the brain used to control the device. The paper by Rezeika et al. [7] in this special issue presents a thorough overview of the main EEG-based spellers that have been developed in the current decade (Jan 2010–Jan 2018). The authors propose a taxonomy based on the type of neural activity exploited: P300, steady-state visual-evoked potentials (SSVEP), motor imagery (MI), or hybrid. They further categorize the spellers based on operation, selection, stimuli modality, gaze dependency, and word prediction, also highlighting the need of keeping the final users in the loop when testing new BCIs. We hope this review will serve as a reference point for researchers interested in the area of BCI-mediated communication. 

Given the importance of spellers in BCI research, it is not surprising that another paper in this special issue focuses on this. One of the most common limitations of BCI spellers is that they are typically tested with able-bodied users, but then fail when tested with locked-in patients. Tonin and colleagues [8] propose a novel BCI speller, that potentially enable patients in the complete locked-in state to express their thoughts, needs, and desires. This speller does not rely on letter-by-letter spelling. Instead, the speller is based on yes/no questions, aimed at gradually restricting possible interpretations and eventually allowing guessing the sentence that the patient would like to spell. The binary answers of the patient are decoded from his/her brain signals, recorded using functional neural infrared spectroscopy (fNIRS). Thanks to an artificial neural network and a binary decoding together with a sequence of questions, this BCI achieves higher accuracy than other BCI spellers.

Many BCI applications, starting from spellers, are based on event-related potentials (ERPs) recorded with EEG. It is, therefore, vital to be able to identify those ERPs (e.g., the P300) from the raw EEG signal recorded from the user’s scalp. The third article of this special issue, by Ramele and colleagues [9], reviews the main methods used for detecting patterns in the EEG activity that could be used in a BCI. The authors compare different methods on both a pseudo-real dataset and the public dataset BCI competition II, both based on (again) a P300-based BCI speller. The authors conclude that fully-automated solutions for identifying such patterns are often suboptimal, and that hybrid systems, using both machine-learning algorithms and the experience of clinicians, may allow BCIs to reach higher accuracies.

The final two articles of this special issue focus on novel applications of BCIs for human augmentation. Nayak and colleagues [10] explore the possibility of detecting changes in human performance, as temperature changes in a work environment, from brain signals. In their study, they have monitored EEG, skin temperature, and heart rate while users were undertaking some office tasks of different difficulty level (i.e., arithmetic problem-solving and typing). They used the room temperature as an independent variable to change the performance of the users in the task, as people are more efficient when put in a comfortable environment. Then, they used neural and physiological signals separately to predict the performance of the user. Weak correlation was found between either the heart rate or the skin temperature and performance level. However, Nayak and colleagues found that EEG features in the power spectrum make good predictors of the performance level of the user. These findings could lead to the development of closed-loop, passive BCIs [11] able to monitor workers and adjust in real time the environmental conditions to maximize their performance.

The last article of this special issue proposes a novel paradigm for integrating humans and machines. In the future, it is very likely that many tasks will be performed by artificial intelligence (AI), but it is also extremely likely that in many other complex tasks there will be a tight integration between humans and AI devices. To achieve the latter, Marc Cavazza [12] proposes to use a BCI to keep the human in the loop, using his/her brain signals to influence the internal heuristic searches performed by the AI devices: the main computations are still performed by AI, with the human, however, being able to supervise the task. The BCI measures the variations of prefrontal asymmetry from a baseline and uses a mapping algorithm to translate such changes into weighting coefficients for the AI device. This framework could potentially be applied to many human–AI problems. 

We hope the readers will find the articles in this special issue interesting and useful. Finally, we would like to thank all the authors who contributed to this special issue, the reviewers for dedicating their time and providing constructive feedback to the submitted papers, and the editorial staff of *Brain Sciences* for their support.
